# Integrating Multiple Data Sources Improves Abundance Estimation in Spatial Capture–Recapture Models

**DOI:** 10.1002/ece3.74413

**Published:** 2026-09-28

**Authors:** Georgina Whittome, John Calambokidis, Philip S. Hammond, Ladd Irvine, Bruce R. Mate, Daniel M. Palacios, Sophie Smout, Chris Sutherland

**Affiliations:** ^1^ Sea Mammal Research Unit, Scottish Oceans Institute University of St Andrews St Andrews UK; ^2^ Cascadia Research Collective Olympia Washington USA; ^3^ Centre for Research Into Ecological and Environmental Modelling, the Observatory, Buchanan Gardens University of St Andrews St Andrews UK; ^4^ Marine Mammal Institute Oregon State University Newport Oregon USA; ^5^ Department of Fisheries, Wildlife, and Conservation Sciences Oregon State University Corvallis Oregon USA; ^6^ Center for Coastal Studies Provincetown Massachusetts USA

**Keywords:** blue whale, data integration, estimating abundance, photo‐identification, spatial capture–recapture, telemetry

## Abstract

Reliable estimates of abundance are fundamental to conservation assessments and measuring the success of conservation actions, especially where populations are in decline or recovery. Here we present an approach that improves statistical precision and biological value of estimates of population size by integrating multiple data sources in a marine system. Using the eastern North Pacific blue whale ( *Balaenoptera musculus* ) as a case study, we apply spatial capture–recapture methods to estimate abundance using photo‐identification data from surveys with known effort. We then integrate opportunistic photo‐identification data and telemetry data, separately and then together, to quantify the value of auxiliary data for generating more robust estimates. The telemetry data sampled a broader area and revealed a very different pattern of space use in the population when compared to the photo‐identification data, which were predominantly collected in higher whale density areas. Thus, the inclusion of these data provides a more representative sample leading to more robust estimates of abundance. Integrated models also improved precision in abundance estimates. The best improvement came from the model integrating all three datasets, but that was largely driven by the addition of the opportunistic photo‐identification data. Addition of opportunistic photo‐identification data led to a 46% reduction in the coefficient of variation when compared to the photo‐identification data from surveys alone. Integrating multiple data streams improves precision of population size estimates, which is critical to accurately track the impact of stressors and to guide management strategies. Making observations to provide robust estimates of abundance can be challenging, especially in wide‐ranging and/or cryptic species where data are often limited despite allocation of significant resources. This study identified data integration as a useful tool to maximise inferences from existing datasets and guide future data collection, informing management planning and conservation policy in the marine setting.

## Graphical abstract

This study demonstrates how integrating multiple data streams improves abundance estimation for a wide‐ranging marine population, eastern North Pacific blue whales. The application of this approach is valuable to accurately track population size which is critical to guide conservation and management strategies. The approach illustrates the value of capitalising on diverse data streams in general, but also in marine systems where data are often limited and integrated data approaches are less common.

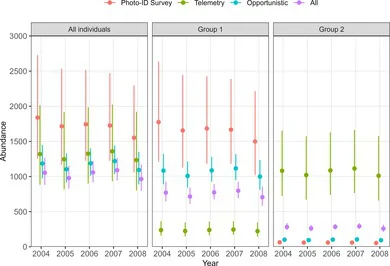

## 1 Introduction

Conservation assessment and quantifying the success of conservation actions rely on robust information on population size. Accurate and precise estimates of abundance are particularly important to monitor the recovery of populations which suffered past exploitation (Callaghan et al. 2024; Magera et al. 2013) or to assess future viability of populations that face increasing anthropogenic pressures (Pirotta et al. 2019; Stewart et al. 2025). Collecting enough data to estimate abundance can be logistically challenging and expensive, especially for wide ranging and/or cryptic species (Moussy et al. 2022). This can be compounded if their environment is difficult to access, for example, marine ecosystems, and in these circumstances, data may be sparse (Hammond et al. 2021; Pal et al. 2022). Maximising insights from often disparate data streams is increasingly critical to address global ecological challenges.

Integrating multiple data sources is recognised as offering significant inferential gains relative to learning from single data streams (Badger et al. 2024; Linden et al. 2018; Sollmann, Gardner, et al. 2013). Methodological developments have begun to make this a viable approach, even with disparate data sources, although integration of data from sampling methodologies that assume different processes and scales require careful interpretation (Bassing et al. 2022; Isaac et al. 2020; Phillips et al. 2019). Combining data sources can increase spatial and/or temporal coverage, providing better representation of population space use and distribution, and hence reducing bias and leading to more precise abundance estimates (Linden et al. 2018; Royle et al. 2013; Tenan et al. 2017). To direct resources most efficiently, knowing which data types are most useful to representatively sample populations and subsequently estimate abundance is highly informative (Boyd and Punt 2021; Luan et al. 2021).

Spatial capture–recapture (SCR) (Tourani 2022) extends traditional capture–recapture methods, adding information about capture locations to account for sources of heterogeneity by modelling imperfect detection (Borchers and Efford 2008; Efford 2004), and in doing so reducing bias in estimates of population size (Royle et al. 2018; Tourani 2022). Data on individuals for SCR studies can come from a range of sources, for example, images from camera trapping (Alexander et al. 2015; Royle et al. 2009), DNA from scat (Sutherland et al. 2018; Thompson et al. 2012) or hair snares (Gardner et al. 2010), photo‐identification surveys (Whittome et al. 2025), and acoustic identifiers (Romeu et al. 2024; Stevenson et al. 2021).

Recent developments of SCR methods allow for the integration of multiple data streams which can provide valuable information on animal space use (Linden et al. 2018; Royle et al. 2013; Tenan et al. 2017). Applications of these integrated models have almost universally demonstrated that additional data leads to improved statistical precision, and in some cases supports increased model complexity (Dupont et al. 2022). Examples of datasets which have been combined with traditional SCR data include telemetry data (Furnas et al. 2018; Linden et al. 2018; Royle et al. 2013), opportunistic photo‐identification data (Leeman et al. 2025; Tenan et al. 2017), binary detection data where individuals are not uniquely identified (Ferreras et al. 2021; Jiménez et al. 2022) and counts from structured survey data (Chandler and Clark 2014), as well as combining multiple types of traditional SCR data (e.g., hair snares and scat, Gopalaswamy et al. 2012; Sollmann, Gardner, et al. 2013, Sollmann, Tôrres, et al. 2013).

Most applications of standard SCR models have been in terrestrial systems, and while there are a growing number of studies in marine systems, few have taken an integrated approach (Winton et al. 2023). Collecting comprehensive data for large marine species is challenging and working in the marine environment requires significant resources for suitable research platforms (Hammond et al. 2021). The huge ranges (thousands of kilometres) of some species mean even long‐term and/or wide‐scale surveys have low encounter rates, exacerbated by the fact that animals are only available (i.e., at the surface) for short periods of time (Schliep et al. 2024; Sequeira et al. 2025). Consequently, in a marine system it is particularly beneficial to maximise insights from data that already exist to inform conservation assessments.

For baleen whales (Mysticeti), methods to obtain robust estimates of abundance are critical, because many populations have been severely depleted by historic commercial whaling (Clapham et al. 1999; Rocha et al. 2014; Stewart et al. 2025). While some populations have demonstrated remarkable recoveries (Calambokidis et al. 2019; George et al. 2004; Seyboth et al. 2023), others remain threatened as anthropogenic threats inhibit recovery (Gabriele et al. 2022; Pettis and Hamilton 2025) or lack the data to quantify their population status (Austin 2021; Thomas et al. 2016). Many taxa face equivalent challenges to baleen whales, so there is a strong need for methods to support population monitoring and assessment. In this manuscript, we integrate three typical wildlife monitoring data types in an SCR modelling framework and, using a baleen whale as a case study, investigate the impacts of data integration on estimates of population size for a wide‐ranging marine species. Specifically, we ask whether data integration improves ecological inferences in terms of statistical precision and biological information, and we discuss this in the context of conservation policy and action.

## 2 Materials and Methods

The goal of our work is to assess the applicability of data integration as a useful tool for generating robust and informative estimates of abundance. To do this, we first introduce the general formulation of SCR, then discuss the integration of two additional non‐standardised data streams. Finally, we demonstrate this data integration approach with a case study where we assess the value of these different data sources in a marine setting where data sparsity is a persistent challenge.

### 2.1 SCR Model Definition

We implement a multi‐session maximum likelihood SCR model which is hierarchical with two sub‐models: an ecological process model and a conditional observation model (Borchers and Efford 2008; Efford 2004; Royle and Young 2008). Individual capture‐recapture data (such as photo‐identification data, henceforth ‘photo‐ID’), y, inform the observation model, which describes individual detection based on a baseline detection rate $\lambda_{0}$ and a spatial scale parameter σ. This is conditional on a spatially explicit Poisson point process model that details the spatial distribution of individual latent activity centres $s_{i}$. The model is an integrated likelihood method, integrating over all possible locations of each individual's (latent) activity centre within the state space (S) to estimate density (Borchers 2012).

In this multi‐session model formulation, each session (year) is independent and within this session, the population is assumed to be closed. The observed encounter data y, are summarised as counts of each individual i at each trap j, where a trap is a location where observations can be collected. The observations are assumed to follow a Poisson distribution (Royle et al. 2014):

$$y_{\mathrm{ij}}\sim \text{Poisson}\left(\lambda_{\mathrm{ij}}\right)$$

We assumed a hazard half‐normal detection function, which arises from the assumption that space use can be approximated by a bivariate normal distribution centred on the individual's activity centre. Under this model, the rate (λ) of detecting an individual i at trap j given the individual's activity centre $s_{i}$ is a decreasing function of the Euclidean distance d between the individual's activity centre $s_{i}$ and the location x of trap j ($x_{j}$):

$$\lambda_{\mathrm{ij}}|s_{i}=\lambda_{0}\exp\left(-\frac{d{\left(x_{j},s_{i}\right)}^{2}}{2\sigma^{2}}\right)$$

$$g_{\mathrm{ij}}|s_{i}=1-\exp\left(-\lambda_{\mathrm{ij}}\right),$$

where the baseline detection rate *λ*_0_ is the detection rate at an individual's activity centre $s_{i}$, and the spatial scale parameter σ determines how quickly detection rate declines with distance from the activity centre. $g_{ij}$ is the probability (hazard) of making a detection of an individual i at a trap j given the individual's activity centre $s_{i}$ at the same distance which is deterministically related to the half‐normal detection function.

The spatial information in the detections simultaneously informs the detection parameters (σ and $\lambda_{0}$) and the Poisson point process model that describes the spatial distribution and intensity of the activity centres. Activity centres are latent (i.e., unobserved) and as such, SCR integrates over all potential activity centres—this integration, therefore, requires the definition of space called a state space or mask (S). The mask is a grid, where each point is the centroid of a pixel. The extent of the point array is buffered around the traps to encompass all plausible activity centres of observed individuals (Royle et al. 2014).

In the simplest version of the SCR, each parameter is described by a single value, but these parameters can also be treated as a function of covariates (Efford 2025b). The parameters of this model ($\lambda_{0}$, σ and density) can be treated as log‐linear regressions with an intercept and, if required, a covariate‐specific slope which can be the same or different between parameters, that is, in the same way as one would apply generalised a linear model (GLM) with a log link function.

The SCR model can, therefore, be described as:

$$\log\left(\lambda_{0,i}\right)=\alpha_{0}+\alpha_{1}x_{\underline{1}}+\ldots +\alpha_{k}x_{k}$$

$$\log\left(\sigma_{i}\right)=\beta_{0}+\beta_{1}x_{\underline{1}}+\ldots +\beta_{k}x_{k}$$

$$\log\left(D_{p}\right)=\delta_{0}+\delta_{1}x_{\underline{1}}+\ldots +\delta_{k}x_{k}$$

where $\lambda_{0,i}$ is the rate of detection of an individual activity centre, with an intercept of $\alpha_{0}$ and $\alpha_{1-k}$ are slopes relating $\;\lambda_{0,i}$ to any number of covariates (i.e., $x_{\underline{1-k}}$). $\;\lambda_{0}$can be adjusted to account for effort, that is, how frequently each trap was surveyed. Similarly, $\sigma_{i}$ is a linear regression representing the rate at which detection declines with distance from the activity centre and density D, which is the expected number of individuals in a grid square or pixel p, can also be modelled as a log‐linear regression. Density can be modelled using a combination of covariates which can be fixed or vary spatially. The total abundance in the state space can be derived by summing the pixel specific abundances (calculated as the pixel specific density * size of the pixel) over all the pixels in the mask. In this study we report abundance as ‘realised’ abundance, which is the sum of individuals observed and those predicted to be present under the model (Efford and Fewster 2013).

### 2.2 Data Integration

Here we describe two common sources of data that lend themselves to integration in an SCR framework, telemetry data and opportunistic photo‐ID data. We note that this study assumes estimates from the single data stream SCR model are reliable, as SCR is an established approach (Bischof et al. 2020; Reich and Gardner 2014; Whittome et al. 2025). Therefore, measures of uncertainty under model extensions with the addition of other data sources can be compared assuming satisfaction in the original estimates.

***Telemetry data*** generate information on an animal's location over time and are the most common type of data to be integrated in SCR (Linden et al. 2018; Patterson et al. 2017; Royle et al. 2013). Tags are affixed to individual animals and can provide location information at a much higher frequency that traditional SCR data collection methods. This offers detailed insights into animal space use which can be used to inform the spatial scale parameter (σ), generally increasing its precision. Unlike most traditional capture‐recapture data used in SCR models, telemetry data are not tied to specific traps, so provide additional information on space use which in turn informs inferences about density (Linden et al. 2018). Note that telemetry data have no associated measure of effort.

Despite the differences in the traditional capture‐recapture data and telemetry data sources, which make the likelihood of observing an animal different in each dataset, the underlying drivers of spatial variation are assumed to be the same (Dupont et al. 2022). Thus, the spatial scale parameter (σ) and activity centres ($s_{i}$) are shared between capture‐recapture data and telemetry data so these parameters in the bivariate normal model can be directly related to the hazard half‐normal detection function and, consequently, the likelihood jointly modelled.

***Opportunistic photo‐ID** data* are typically unstructured photo‐ID encounters at known locations and times, but with no measure of effort associated. Where there are multiple opportunistic data points for an individual, they are at an irregular frequency. Therefore, they can be considered equivalent to telemetry data from sporadically operating tags, in that they provide the same information but usually with far fewer data points. For the purposes of this study opportunistic data are treated as equivalent to telemetry data.

### 2.3 Application in Eastern North Pacific (ENP) Blue Whales ( *Balaenoptera musculus* )

Whittome et al. (2025) applied SCR methods to photo‐ID data from ENP blue whales collected on dedicated surveys with associated effort and established SCR as a viable method to estimate abundance for this population. From 2004 to 2008, there are also opportunistic photo‐ID data and telemetry data from animal‐bourne tags available. This offers the opportunity to look at the effects of data integration on abundance estimates in the SCR framework, which could be applicable to many populations. The focus of this manuscript is evaluating the estimates from the different data types relative to each other to understand the effects of data integration. The extent to which these estimates inform ENP blue whale abundance estimates more generally is considered in Discussion.

ENP blue whales typically migrate north from waters south of the United States West coast (USWC) in spring/early summer, spending the summer and autumn feeding off the USWC and then migrate south again in late autumn/winter (Bailey et al. 2009; Busquets‐Vass et al. 2020; Mate et al. 1999). This application estimates abundance of ENP blue whales off the USWC each year, so we selected data from June to November, the main months for ENP blue whale presence in this area.

### 2.4 Data Collection and Preparation

***Dedicated photo‐ID survey data*** of blue whales were available from Cascadia Research Collective (CRC) small boat surveys along the USWC (Calambokidis et al. 2009; Calambokidis and Barlow 2004, 2020). These photo‐ID surveys aimed to provide broad spatial and temporal coverage along the coastline, while maximising encounters with blue and humpback ( *Megaptera novaeangliae* ) whales and usually extended no further than 50 km from the daily launch point (Calambokidis et al. 2009). Photo‐ID images underwent processing, including quality assessment and matching, before inclusion in a database (see Supporting Information for additional detail).

The north–south extent of the data collection was the length of the USWC, so effective ‘traps’ were generated by overlaying a grid onto all blue whale photo‐ID survey encounters in CRC database with a cell size of 0.4°, approximately 44 km, where the centroid of the grid cell became the ‘trap’ (Whittome et al. 2025). We tested a range of grid cell sizes from 0.05° to 0.7° and 0.4° allowed us to maximise the number of spatial captures while minimising computation (as detailed in Whittome et al. 2025). This cell size was well below the range exhibited by ENP blue whales as identified by tag data, which ranged from a minimum of 12° latitude in July to 52° degrees latitude in November (Fuller et al. 2016; López‐Bao et al. 2018; Milleret et al. 2018; Palacios et al. 2025). The state space or mask (S) is a grid, where each centroid of a pixel was set at 10 km apart. The extent of the point array was buffered around the traps to encompass all plausible activity centres of observed individuals, in this case to 200 km except the eastern edge which was clipped with a polygon of the coastline (Figure 1) (Royle et al. 2014; Whittome et al. 2025).

Each encounter was allocated to its closest trap. For this analysis, we selected only animals with right side images to avoid duplication (Galletti Vernazzani et al. 2017; Whittome et al. 2024). Additionally, to maintain temporal independence, we thinned the data so that each animal was only recorded once per trap per calendar week. The capture history was then constructed with each year as a single sampling session. Detection data were summarised as the total number of independent detections (encounters) each session (year) (Figure 1).

**FIGURE 1:**
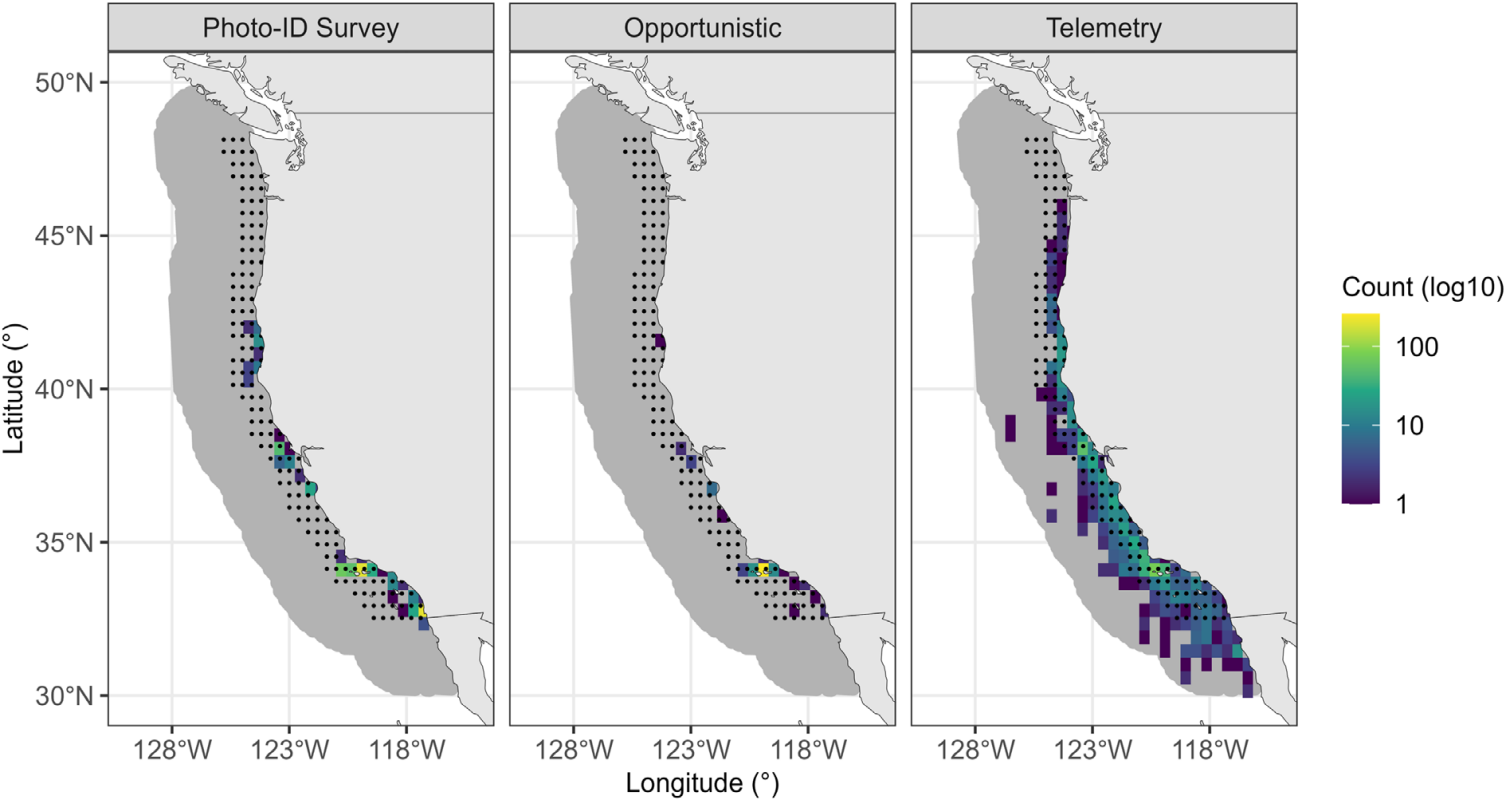
Maps of study area, trap array and counts of encounters by data type after processing. Grey shaded area represents the state space, known in SCR as a mask. Black points are trap locations spaced at a resolution of 0.4°, approximately 44 km. Shaded coloured boxes represent the number of encounters in that area by data type.

Effort data associated with the photo‐ID images were only available at the daily summary level, so we assumed any traps within 50 km north or south of the daily launch point were ‘on effort’ for that day (see Figure S1). To align with the weekly independence assumption in the detection data, we calculated the number of calendar weeks each latitudinal band was surveyed. $\;\lambda_{0}$ is the per week detection rate for each trap, which was adjusted based on how many weeks a trap was sampled.

***Opportunistic photo‐ID data*** (‘opportunistic data’ from here) were also extracted from the CRC database (Figure 1). Third parties routinely share blue whale photo‐ID images with CRC, including whale‐watching organisations, scientific researchers and nature photographers. Between 2004 and 2008, 93% of opportunistic photo‐ID images were collected in the Santa Barbara Channel (Figure 1). These images and the resulting capture histories were processed using the same protocol as the CRC photo‐ID survey images, including thinning to maintain temporal independence.

Initially, we modelled all individuals in the opportunistic data but found that this led to unrealistically large abundance estimates, because most individuals were encountered only once with no associated effort (see Figure S2). Therefore, we only retained individuals that were identified in the same session (year) in both the dedicated photo‐ID survey and opportunistic data, thus adding additional detection information to known individuals.

***Telemetry data*** were collected by Oregon State University from satellite‐linked radio tags deployed on blue whales predominantly during summer and autumn off central and southern California (Irvine et al. 2025; Mate et al. 1999). Details of tag programming and deployment are described in Palacios et al. (2025). Argos locations from tags are usually at irregular intervals and have associated error (Irvine et al. 2020), so tracks were regularised using a state space model (see Palacios et al. 2025 for detail of the model fitting). The outputs from these state space models were three location points per day for each tag deployment. To fit with our analysis period, data were limited to June to November in 2004 to 2008. Only data for individuals which could be matched to the CRC photo‐identification database were used to ensure there was no duplication of individuals or cases where the same individual appeared in both datasets without being linked.

The length and timing of tag deployments meant that often these included migratory movements, so we filtered the data based on categorisations of behavioural state from the state space model to isolate space use off the USWC as opposed to migratory movement. For each individual, we identified the first and last area restricted search (ARS) behaviour without the whale leaving the state space. Even if there was transit behaviour between the ARS periods, we assumed this to be normal movement or search behaviour on the feeding grounds. If there was more than one period, we selected the longer one (see Supporting Information for further detail). We note that photo‐ID data from surveys and opportunistic sources were not treated in the same way and may include migratory points. However, if an animal was seen while migrating along the USWC, it is equally likely to be seen while moving north or south which we assume would minimise bias. Most of the telemetry deployments were in summer, from late July onwards when the whale had arrived on the USWC feeding ground, so the migration north to the USWC was not recorded. However, the southerly migration later in the year would be included when tags stayed on long enough. This created a potential imbalance, hence the need to remove transit periods at the end of the deployments. Additionally, the photo‐ID data have far fewer points per individuals so the impact of including a single point during migration would be minimal.

We applied the same independence assumptions to the telemetry data, as to the dedicated photo‐ID survey and opportunistic data. We thinned the observations, so that an individual was only recorded once in a trap in any week. This way we retained the spatial information but also aided model fitting by reducing the number of points. As for the opportunistic data, these data were used to inform detection in the observation sub‐model.

We tested outcomes of four different data combinations, which henceforth are referred to as the variant name in brackets:

1. Photo‐ID survey only (Photo‐ID survey)
2. Photo‐ID survey + opportunistic photo‐ID (Opportunistic)
3. Photo‐ID survey + telemetry (Telemetry)
4. Photo‐ID survey + opportunistic photo‐ID + telemetry (All)

### 2.5 SCR Model Application

We ran all models in R ver. 4.4.3 (R Core Team 2025) using package **secr** (Efford 2025a). We implemented the model described in the SCR model definition with covariates applied to each of the parameters. To account for heterogeneity in detection which has previously been identified in the ENP blue whale photo‐ID data, we applied a fixed 2‐class finite mixture model to both detection parameters, σ and λ (Efford and Mowat 2014; Pledger 2000; Pledger and Phillpot 2008; Whittome et al. 2025). In this formulation both σ and λ have two parameters, an intercept and the difference in detection between the two latent mixture groups. We also applied a linear trend over time to the density parameter. We modelled the density of whales per 10 km^2^, as a log‐linear regression with an intercept and a trend in session (year) at each pixel in the mask.

The different data collection methods represent space use in very different ways despite describing the same underlying ecological process, leading to differences in σ between the datasets which makes modelling a joint likelihood challenging and can lead to non‐convergence. To address this, we used the scaling feature ‘telemetryscale’ with the secr.fit function (Efford 2024). Model‐fitting failure can be caused by the near zero contribution of the telemetry data to the joint likelihood, which is common in large datasets where observed locations may appear unlikely; even moderately small probabilities can then multiply to values near zero in the likelihood. To address this, the telemetry data were scaled to larger numbers allowing the model to converge (Efford 2025b). During model development, finding appropriate start values was an essential step, which was achieved by a combination of adding portions of data at a time and increasing the value of the ‘telemetryscale’ feature. For the Opportunistic variant we used start values from the Photo‐ID survey variant, and for the All variant we used start values from the Opportunistic variant. For the Telemetry variant, even with the use of ‘telemetryscale’ there were convergence issues when using the Photo‐ID survey variant as start values. To address this, we added the telemetry data in two equal portions, using start values from one model in the next. This does not affect inference or parameter values but is a useful technical step to facilitate model fitting.

## 3 Results

Over the 5‐year period, from 2004 to 2008, a total of 486 days of photo‐ID survey effort, averaging 97 per year, generated 767 encounters of 452 individual blue whales (Figure 1, Figure S1). In 468 of the encounters, the individuals were only encountered once within the same year. There were 314 opportunistic encounters of 149 individuals (Figure 1). A total of 37 tags were deployed on unique individual whales, from which 1014 location points were derived (Figure 1).

Model parameters revealed some distinct differences between the model variants. Table 1 describes the real estimates (see Supporting Information for beta parameter estimates, Figure S3). In summary, the parameter values support the inclusion of the mixture parameter (*p*_mix_) as it showed distinct differences in detection between the two groups. Compared to group 2, group 1 had a much higher baseline detection rate but much smaller values of σ, so hereafter group 1 will be referred to as ‘narrower ranging’ and group 2 as ‘wider ranging’. The telemetry variant was predominantly made up of individuals in the wider ranging group, whereas all other models were predominantly made up of individuals in the narrower ranging group. Overall, density and abundance were stable across the study period and the addition of data led to improved precision in estimates of density and abundance.

**TABLE 1:** Summary of spatial capture–recapture (SCR) model coefficients and associated variance transformed onto the real scale for each of the four model variants.

Parameter	Model variant	Estimate	Lower CI	Upper CI
*P*_mix_	Photo‐ID survey	0.035	0.005	0.207
	Opportunistic	0.085	0.052	0.136
	Telemetry	0.820	0.687	0.904
	All	0.268	0.176	0.385
Baseline encounter (λ) group 1	Photo‐ID survey	0.095	0.072	0.126
	Opportunistic	0.713	0.580	0.878
	Telemetry	0.502	0.384	0.655
	All	0.541	0.437	0.670
Baseline encounter (λ) group 2	Photo‐ID survey	0.005	0.001	0.018
	Opportunistic	0.028	0.015	0.052
	Telemetry	0.005	0.002	0.013
	All	0.014	0.007	0.026
Encounter rate change (σ) group 1 (km)	Photo‐ID survey	17.4	15.9	19.0
	Opportunistic	13.3	12.5	14.1
	Telemetry	26.9	24.9	29.0
	All	17.0	16.1	17.9
Encounter rate change (σ) group 2 (km)	Photo‐ID survey	449	291	692
	Opportunistic	175	147	208
	Telemetry	174	149	204
	All	167	149	186
Density (D) (whales/10 km^2^)				
2004 (intercept)	Photo‐ID survey	0.026	0.017	0.039
	Opportunistic	0.017	0.013	0.020
	Telemetry	0.018	0.012	0.028
	All	0.015	0.012	0.018
2005	Photo‐ID survey	0.025	0.017	0.037
	Opportunistic	0.017	0.014	0.020
	Telemetry	0.018	0.012	0.028
	All	0.015	0.012	0.017
2006	Photo‐ID survey	0.024	0.017	0.035
	Opportunistic	0.016	0.014	0.020
	Telemetry	0.018	0.012	0.028
	All	0.015	0.012	0.017
2007	Photo‐ID survey	0.024	0.016	0.034
	Opportunistic	0.016	0.014	0.020
	Telemetry	0.018	0.012	0.028
	All	0.014	0.012	0.017
2008	Photo‐ID survey	0.023	0.016	0.033
	Opportunistic	0.016	0.013	0.020
	Telemetry	0.018	0.012	0.028
	All	0.014	0.012	0.017

*Note:* Lower CI is the lower 95% confidence interval, and upper CI is upper 95% confidence interval. Parameters are density (*P*_mix_), the probability that a randomly selected individual is in group 2, baseline encounter rate (λ) for each group, the spatial parameter (σ) that determines the distance over which encounter rates reduce towards 0 for each group, and density (D) for each year of the study, where the first year 2004 is the intercept and subsequent years are a function of a linear trend in Session (year).

When modelling detection, we applied a 2‐class mixture parameter to $\;\lambda_{0}$ and σ to model unobserved heterogeneity and account for possible bias in abundance estimates (Pledger 2000; Pledger and Phillpot 2008). To achieve this, the mixture parameter allowed capture probability and likelihood of detection to be modelled as two separate classes (Pledger 2000). Membership in these latent classes varied by model, as did the uncertainty around the estimate. The chance of an individual being randomly allocated to the wider ranging group (*P*_mix_) was very low (< 10%) for the Photo‐ID survey and Opportunistic variants (Table 1). The Telemetry variant showed the opposite with an 82% chance of random allocation to the wider ranging group. The influence of the telemetry data meant that the chance of random allocation to the wider ranging group in the All variant was 27%, slightly higher than the Photo‐ID survey and Opportunistic variants.

The baseline encounter rates ($\lambda_{0}$) varied by group but the patterns were consistent between the groups, with the lowest values for the Photo‐ID variants followed by the Telemetry, All and Opportunistic variants (Table 1). Overall, values were much lower for the wider ranging group, ranging from 0.005 for the Photo‐ID survey variant to 0.028 for the Opportunistic variant. For the narrower ranging group, the lowest value was for the Photo‐ID variant at 0.095 but much higher (> 0.5) for the three other variants, peaking at 0.713 with the Opportunistic variant.

The rate at which detection declines as animals move away from the activity centre, a measure of their space use (σ), was much lower for the narrower ranging group than the wider ranging group by an order of magnitude (Table 1). For the narrower ranging group, the lowest value was 13.3 km for the Opportunistic variant, and highest for the Telemetry variant at 26.9 km. For the wider ranging group, the All, Telemetry and Opportunistic variants had similar values around 170 km, but the Photo‐ID survey variant had a much larger value of 448.6 km.

The density intercept, that is, in the first year of the study (2004), was largest but had the most uncertainty in the Photo‐ID survey variant (Table 1). Density and associated uncertainty decreased in the Telemetry, Opportunistic and All variants. The linear trend over time showed a minimal decrease year on year (Table 1). For the Density Session trend parameters, the beta coefficient 95% confidence intervals (CI) spanned 0 for every model suggesting no significant decline (Figure S3) which is also evident in the annual density estimates (Table 1).

Consequently, abundance showed no marked trend over time (Figure 2). Abundance estimates were highest for the Photo‐ID survey variant followed by the Telemetry, Opportunistic and All variants. In all years, 95% CI were widest in the Photo‐ID survey variant followed by the Telemetry, Opportunistic then All variants (Table 2). However, there is variation in that pattern in the relative uncertainty; the coefficient of variation (CV) is highest in the Telemetry variant, followed by the Photo‐ID survey, Opportunistic then All variants (Table 2).

**FIGURE 2:**
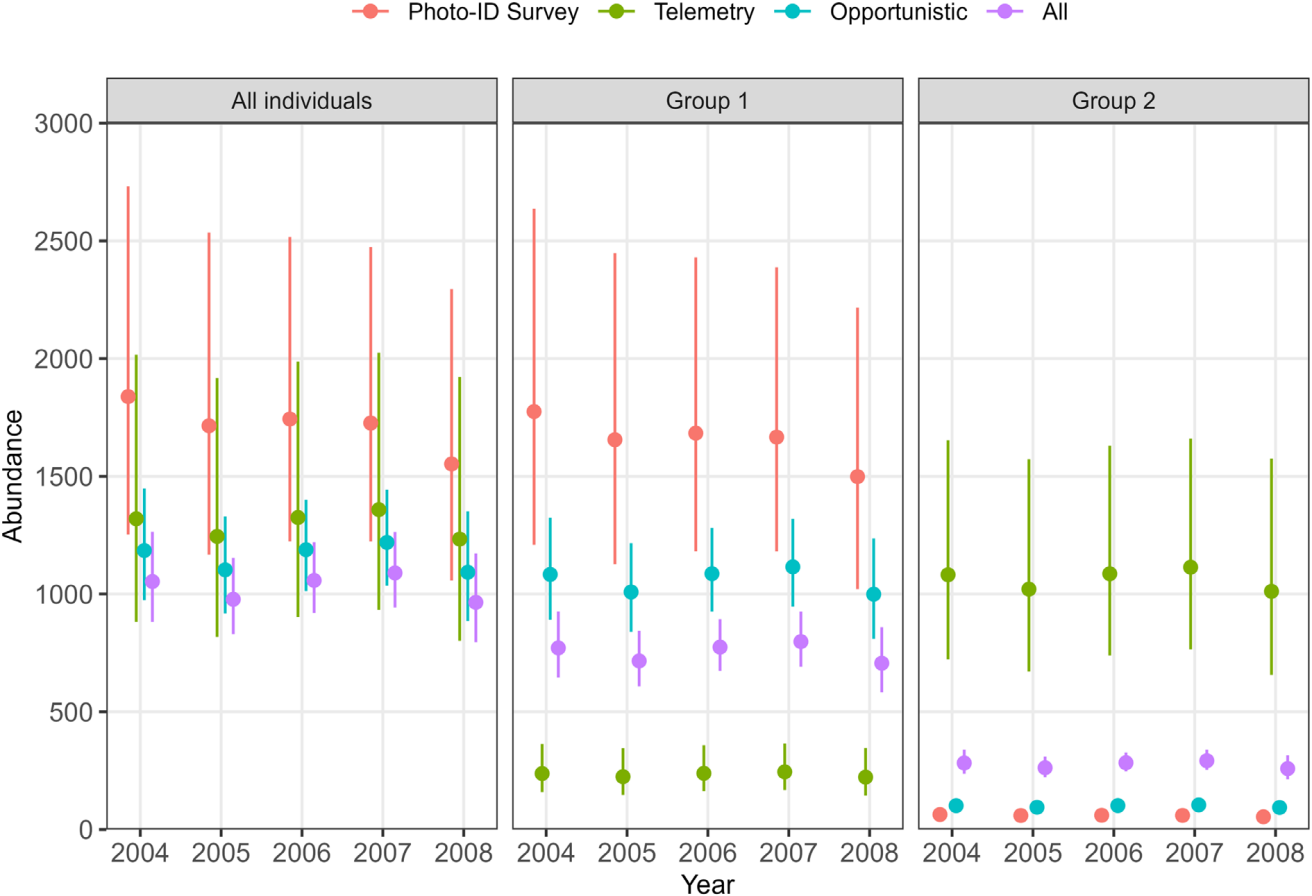
Abundance estimates left: All individuals, centre: Latent group 1 (narrower ranging) as determined by mixture parameter, right: Latent group 2 (wider ranging) as determined by mixture parameter. Points are maximum likelihood estimates and vertical lines are the 95% confidence intervals.

**TABLE 2:** Summary of abundance estimates and associated confidence metrics by model.

		Model			
		Photo‐ID survey	Opportunistic	Telemetry	All
2004	N	1838	1185	1320	1053
	CI	1479	474	1135	384
	CV	0.202	0.102	0.215	0.092
2005	N	1715	1103	1245	977
	CI	1369	412	1100	323
	CV	0.200	0.095	0.221	0.084
2006	N	1743	1188	1325	1058
	CI	1293	388	1085	300
	CV	0.186	0.083	0.205	0.072
2007	N	1726	1219	1358	1090
	CI	1250	408	1092	321
	CV	0.182	0.085	0.201	0.075
2008	N	1552	1092	1233	965
	CI	1238	465	1120	377
	CV	0.200	0.108	0.227	0.099

*Note: N* represents total abundance, CI is the width of the 95% confidence interval described as number of whales, and CV is the coefficient of variation.

The combination of the estimates of the various model parameters for each group resulted in marked differences among the models in estimates of the abundance of animals in the two mixture groups (Figure 2). Abundance from the Telemetry variant contributed most of the individuals to the wider ranging group and provided a minimal contribution to the narrower ranging group. Conversely, the Photo‐ID survey and Opportunistic variants almost exclusively contributed to the abundance in the narrower ranging group. As expected, the All variant sat in between the two but had almost three times more animals in the narrower ranging group than the wider ranging group.

## 4 Discussion

This study explored the integration of photo‐ID data collected from small boat surveys with telemetry and/or opportunistic photo‐ID data. In the integrated models, the spatial scale parameter (σ) and activity centres ($s_{i}$) are modelled using a joint likelihood between the different datasets and therefore represent a weighted combination of the contributing data sources, with their relative influence determined by their respective structure and information content (Jiménez et al. 2022). We show that the addition of data improved precision of abundance estimates, with the opportunistic photo‐ID data having the strongest influence. We also demonstrate that the telemetry data represented a different movement pattern from the photo‐ID datasets providing more representative sampling of the population when combined and offering interesting biological insights into movement patterns. These findings highlight the value of using existing data sources and data integration to learn more about population abundance, and to generate more robust abundance estimates, which can inform future conservation assessment and action.

### 4.1 Representation of Blue Whale Movement

The photo‐ID and telemetry datasets are sampling the same underlying ecological process in very different ways but have been demonstrated to still predict similar distribution patterns (Bassing et al. 2022; Tenan et al. 2017). Photo‐ID data generally represent high density areas with few sightings of many individuals representing a population‐level process. In this case, data were heavily concentrated in coastal waters as they were collected from small boats. Conversely, telemetry data provide many locations of few individuals representing an individual‐level process. These data also sample lower density areas, so are a more complete spatial representation of the area used by a small set of individuals. This variation in sampling is reflected in the 2‐class mixture parameter which was included as heterogeneity in capture probabilities has been previously identified in the ENP blue whale photo‐ID data (Calambokidis and Barlow 2020; Whittome et al. 2025). Inclusion of the 2‐class mixture parameter has been shown to be sufficient to substantially correct for bias in abundance estimates caused by heterogeneity, and is assumed to apply in this case but cannot be verified in the absence of formal goodness of fit tests (Pledger 2000; Pledger and Phillpot 2008). While all data types had individuals attributed to both mixture groups, the photo‐ID datasets were predominantly attributed to individuals in the narrower ranging group and the telemetry data were predominantly attributed to individuals in the wider ranging group, suggesting very different movement patterns. While a small portion of the photo‐ID data represented the wider ranging movement pattern, the addition of telemetry data provided more data on wider scale blue whale movement. Despite this, the integrated model is not necessarily a complete picture of the broader population for two reasons. Firstly, tagged individuals were all tagged in coastal waters, where photo‐ID data were also collected. Thus, while the telemetry data show a broader picture of movement in the tagged animals, there may also be a portion of the population which spends more time offshore which would be less likely to be tagged. Secondly, the tagged animals may not be representative of all the animals in the photo‐ID dataset. They were selected to be tagged based on their availability and suitability for tagging and thus were not a random sample of individuals based on for example, sex and age class. Therefore, the tagged individuals were not necessarily representative of the broader population.

### 4.2 Precision of Abundance Estimates

Data integration led to improved precision of relative (CV) and absolute (width of CI) confidence intervals. The All model variant was the most precise model variant in relative and absolute terms (Table 2). This aligns with previous studies that have found integration of multiple datasets reliably improves precision around estimates even if data sources are dependent (Abadi et al. 2010; Linden et al. 2018; Royle et al. 2013). However, the precision of estimates from the All variant was only slightly better than the Opportunistic variant, suggesting that there was minimal precision gain from the addition of the telemetry data. The Opportunistic model variant was markedly better than the dedicated Photo‐ID survey variant, with a minimum reduction of 46% in the coefficient of variation across all 5 years of the study. This is likely driven by the fact that all individuals in the opportunistic data were also present in the photo‐ID survey data. In increasing the number of recaptures for these individuals, there was more information about detection, specifically these data inform the spatial scale parameter (σ), so this led to an improvement in precision of this parameter and subsequent abundance estimates. This highlights the value of opportunistic photo‐ID sightings to complement survey photo‐ID data collected with known effort.

The telemetry data provided a slight improvement in precision in abundance estimates over the Photo‐ID survey variant in terms of absolute precision but not in terms of CV (Table 2). It could be that the telemetry data are not providing substantial additional precision to the σparameter. For the narrower ranging group, the width of the σ CI for the Telemetry variant was 4.1 km vs. 3.1 km for the photo‐ID survey variant. While the narrower ranging group was only reflective of a small portion of the tagged individuals (18.2%), in relative terms the CI was 27% wider. For the wider ranging group, the width of the σ CI for the Telemetry variant was 55 km vs. 401 km for the photo‐ID survey variant, so the Telemetry variant estimate of σ was much more precise. However, the wider ranging group had much larger space use than and a lower baseline encounter rate ($\lambda_{0}$) than the narrower ranging group, so was inherently less certain about the location of individual activity centres. Consequently, as the telemetry data were predominantly made up of individuals in the wider ranging group, it follows that there might not be the expected precision gains, when the photo‐ID survey variant is mainly represented by individuals with more precise baseline encounter rates ($\lambda_{0}$) and reduced space use (σ).

There is also a technical rationale which could explain the lack of increase in precision in the Telemetry variant based on the way the **secr** package integrates telemetry data. **secr** assumes that tagging an individual is conditional on that individual's activity centre and equivalent to being detected at least once in the photo‐ID survey. This is valid when telemetry devices are deployed during the SCR survey but problematic in cases like our data when the surveys are not concurrent and tagged individuals may not be encountered, and could result in inflated variance (Kodi 2024).

### 4.3 Differences in Abundance Estimates Between Models

Imprecision in the detection parameters can lead to bias in the estimation of the effective sampling area (ESA), a derived quantity that reflects the area effectively covered by the detector array once animal movement and detection probability are accounted for, which includes the space within the buffer around the array (Borchers 2012; Borchers and Efford 2008). While additional data in integrated models only inform the spatial scale parameter (σ), improving the precision of σ can also improve precision of the baseline detection rate ($\lambda_{0}$) because these parameters are estimated jointly from the spatial pattern of detections. This strong coupling means more precise estimation of σ leads to a better‐defined detection function, which in turn improves estimation and reduces bias in the ESA. As abundance (and density) is inferred relative to this ESA, improvements in estimating σ can ultimately propagate through the detection model to influence abundance estimates. Estimation of ESA is also influenced by the spatial extent of detections. As ESA increases, detections inform a larger proportion of the state space, thus providing more information on space use and reducing the need to extrapolate density into poorly informed regions of the mask. When density is assumed constant, this reduction in extrapolation can result in lower overall abundance estimates. This explains why when comparing the four sets of abundance estimates, we observe that estimated abundance changes when more data are incorporated. The ESA increased from 47,583 km^2^ in the Photo‐ID variant, to 64,597 km^2^ in the Telemetry variant, 71,887 km^2^ in the Opportunistic variant and 81,276 km^2^ in the All variant. Integrating the telemetry data increased ESA by expanding the area over which individuals were detected. Integrating the opportunistic data did not expand the spatial extent of sampling, but did result in a larger estimated $\lambda_{0}$, while also improving precision in the detection model. Thus, the estimates from variants with additional data integrated likely provide a more realistic representation of space use, thereby increasing robustness in the resulting abundance estimates.

### 4.4 Biological Interpretation of Abundance Estimates

When integrating multiple data sources, a clear understanding of appropriate biological context and of how model inference might be affected is essential for accurate interpretation of results. The individuals sampled in this study were representative of the coastal USWC as the photo‐ID data were collected in coastal waters. From previous line‐transect surveys we know that some individuals spend time further offshore and, while the ENP is considered a single population, the amount of inshore to offshore mixing is unknown (Barlow 2016). The telemetry data show some offshore use, but all individuals were tagged in coastal waters, so individuals that spend more time offshore are likely underrepresented. In this study, we add more information about the same set of individuals, because almost all individuals in the additional datasets were present in the photo‐ID survey data, but we cannot make inferences about any portion of the population which preferentially uses other areas of the ENP blue whale range.

### 4.5 Applicability of Data Integration for Marine Species

Conservation assessments and measuring the effectiveness of conservation action relies on robust and accurate estimates of population size. We largely reflect on baleen whales, but this approach could be applied to any population under assessment with photo‐ID and telemetry datasets, such as sharks (Porcher and Darvell 2022; Worm et al. 2024). Many baleen whale populations continue to require population size assessment due to varying recovery rates following depletion by commercial hunting (Rocha et al. 2014; Stewart et al. 2025). Most populations also face increasing pressure from anthropogenic activities, including entanglement in fishing gear and ship strikes from large commercial vessels (Rockwood et al. 2018; Tackaberry et al. 2022). Mitigation actions can be taken to reduce the impact of these threats, such as speed limits for ships and fisheries restrictions when whales are present in large numbers. As these threats are expected to increase in the coming years, possibly exacerbated by the effects of climate change, having methods to robustly track population size is crucial (Stewart et al. 2025).

Collecting data to estimate population abundance often requires significant resources. The marine environment usually entails vessel‐based operations increasing the logistical complexity and financial cost, and even major efforts can yield disparate data. Consequently, statistical methods that maximise insights from that data are very valuable, especially with the backdrop of an uncertain and ever challenging research landscape. Often different data types are collected by different groups, so building collaborations across groups may provide further opportunities to combine existing datasets to provide new insights for population monitoring.

Opportunistic data are typically much less costly to collect than using dedicated surveys as they are usually collected through citizen science programmes or provided by commercial organisations (e.g., whale watch companies). These data have limitations, most often, no associated effort about the data collection, and commercial trips are often spatially concentrated to popular tourist locations (Leeman et al. 2025), but this study shows that opportunistic data can be a valuable supplementary resource in population monitoring. Platforms such as Happywhale.com have been developed to gather such data, and so large volumes of opportunistic data can be accessed with relative ease (Cheeseman et al. 2017; Ramos et al. 2023). We consider opportunistic photo‐ID data a valuable resource to supplement other data streams when estimating abundance.

## 5 Conclusions

The availability of three datasets for ENP blue whales provided an opportunity to test the effectiveness of data integration for a wide‐ranging species in the marine environment. The two datasets integrated with the photo‐ID survey data both provided important biological inferences; opportunistic photo‐ID data improved precision of abundance estimates and telemetry data identified a broader space use through the 2‐class mixture parameter. Marine species are logistically challenging and expensive to study so approaches which can maximise insights from existing data sources hold significant value, especially where the results deliver insights not attainable from analysis of the datasets individually. As many marine species increasingly face pressure from anthropogenic threats, statistical approaches that inform population status are essential tools to inform conservation assessment and detect changes in population status.

## Author Contributions

**Georgina Whittome:** conceptualization (equal), formal analysis (lead), methodology (equal), visualization (lead), writing – original draft (lead), writing – review and editing (equal). **John Calambokidis:** conceptualization (equal), data curation (equal), funding acquisition (equal), project administration (equal), supervision (equal), writing – review and editing (equal). **Philip S. Hammond:** conceptualization (equal), supervision (equal), writing – review and editing (equal). **Ladd Irvine:** data curation (equal), project administration (equal), writing – review and editing (equal). **Bruce R. Mate:** funding acquisition (equal), project administration (equal), writing – review and editing (equal). **Daniel M. Palacios:** data curation (equal), funding acquisition (equal), project administration (equal), writing – review and editing (equal). **Sophie Smout:** conceptualization (equal), supervision (equal), writing – review and editing (equal). **Chris Sutherland:** conceptualization (equal), formal analysis (supporting), methodology (equal), supervision (equal), writing – review and editing (equal).

## Funding

The OSU telemetry dataset funding from 1994 to 2008 was provided by the Office of Naval Research (grant Nos. 9610608, 0010085, 0310862), the Tagging of Pacific Pelagics Program of the Census of Marine Life, the National Science Foundation, the National Aeronautics and Space Administration (grant No. NNX11AP71G), the Alfred P. Sloan Foundation, the Moore Foundation, the Packard Foundation, National Geographic Society, and the Marine Mammal Endowed Program at Oregon State University. Funding for the photo‐ID from dedicated surveys was provided by the National Oceanic and Atmospheric Administration under multiple grants and contracts for both the work that generated the data and the analysis.

## Conflicts of Interest

The authors declare no conflicts of interest.

## Supporting information


**Figure S1:** Days of effort from small boat surveys conducted by the Cascadia Research Collective. Days of effort are per year by one degree latitude bands off the United States west coast.
**Figure S2:** Assessment of the impact of adding opportunistic data for individuals not present in the photo‐ID survey data. Individual IDs were randomised and then added to the opportunistic model one at a time. Plots reflect abundance, where each addition of an individual is represented by a different colour. There were 352 individuals present in the opportunistic data not present in the photo‐ID survey data. We were able to add 53 individuals to the model before it failed to converge. The addition of the 53rd individual led to extremely unrealistic abundance estimates (see left plot). Right plot shows increasing abundance with each individual from 1 to 52.
**Figure S3:** Beta parameter estimates from the SCR model. Length of the bar represents the width of the confidence interval around the parameter estimates.

**Data S1:** ece374413‐sup‐0002‐DataS1.zip.

## Data Availability

Data and code are available from the Open Science Framework: https://doi.org/10.17605/OSF.IO/XYKP6. The OSU telemetry dataset is also available from the Movebank Data Repository (https://doi.org/10.5441/001/1.5ph88fk2).
