# Supplementary material for: Integrating Multiple Data Sources Improves Abundance Estimation in Spatial Capture–Recapture Models
Source: Ecol Evol. 2026 Sep 28;16(10):e74413. doi: 10.1002/ece3.74413 (PMC13619746; doi:10.1002/ece3.74413)
Supplement: Supplementary file 1 [file ECE3-16-e74413-s002.docx]

**Supporting Information**

**Data collection and preparation: Further information**

**Photo-identification survey data collection**

Cascadia Research Collective (CRC) have conducted small boat surveys along the United States West coast (USWC) between 32°N and 49°N every year since the late 1980s, predominantly between June and November (Calambokidis et al., 2009; Calambokidis & Barlow, 2004, 2020). Surveys were largely conducted from rigid hulled inflatable boats and usually extended no further than 50km from the daily launch point (Calambokidis et al., 2009). The surveys aimed to provide broad spatial and temporal coverage along the coastline, while maximising encounters with blue and humpback whales. Photo-identification images were taken of the flanks of blue whales, capturing both sides during an encounter if possible. Images were added to the CRC database if the quality was adequate after assessment using a three-tier criterion (Calambokidis et al., 2009). Images were matched based on pigmentation patterns (Gendron & Ugalde De La Cruz, 2012; Sears et al., 1990), with at least two people verifying each match before inclusion in the database. For this analysis, we used encounters of individuals seen between June and November from 2004 to 2008 along the USWC between latitudes 32.53°N and 48.5°N. We selected only animals with right side images to avoid duplication (Galletti Vernazzani et al., 2017; Whittome et al., 2024).

**Telemetry data processing**

While peak number of whales off the USWC coast are always in summer and fall, there can be marked annual variation in the timing of animals arriving and leaving the USWC (Oestreich et al., 2022). Most individuals were tagged off the USWC between late July and mid-September so had completed their Northward migration by the time the telemetry was deployed, but the deployment included the southerly migration. We used categorisations of behavioural state from the state space model to ensure we were excluding migratory movement from the telemetry dataset. Two behavioural modes were classified based on turning angles and autocorrelation in speed and direction (Palacios et al., 2025). Where animals showed more autocorrelation and lower turning angles, tracks were categorised as transiting, where animals had less autocorrelation and higher turning angles, tracks were categorised as area restricted search (ARS), a pattern associated with feeding or searching for food (Palacios et al., 2025). Each telemetry point was given a value between 1 and 2 and then points >1.5 were categorised as ARS and points <1.5 were categorised as transitioning (Palacios et al., 2025). For each individual, we identified the first and last ARS behaviour without the whale leaving the state space. Even if there was transit behaviour between the ARS periods, we assumed this to be normal movement or search behaviour on the feeding grounds. If there was more than one period, selected the longer one.

**Supporting Figures**

**Figure S1.** *Days of effort from small boat surveys conducted by the Cascadia Research Collective. Days of effort are per year by one degree latitude bands off the United States west coast.*

**Figure S2**. *Assessment of the impact of adding opportunistic data for individuals not present in the photo-ID survey data. Individual IDs were randomised and then added to the opportunistic model one at a time. Plots reflect abundance, where each addition of an individual is represented by a different colour. There were 352 individuals present in the opportunistic data not present in the photo-ID survey data. We were able to add 53 individuals to the model before it failed to converge. The addition of the 53^rd^ individual led to extremely unrealistic abundance estimates (see left plot). Right plot shows increasing abundance with each individual from 1 to 52.*

**Figure S3.** *Beta parameter estimates from the SCR model. Length of the bar represents the width of the confidence interval around the parameter estimates.*

**References**

Calambokidis, J., & Barlow, J. (2004). Abundance of blue and humpback whales in the eastern north pacific estimated by capture-recapture and line-transect methods. *Marine Mammal Science*, *20*(1), 63–85. https://doi.org/10.1111/j.1748-7692.2004.tb01141.x

Calambokidis, J., & Barlow, J. (2020). Updated abundance estimates for blue and humpback whales along the US West Coast using data through 2018. *NOAA Technical Memorandum NMFS-SWFSC-634*. https://ntrl.ntis.gov/NTRL/

Calambokidis, J., Falcone, E., Douglas, A., Schlender, L., & Huggins, J. (2009). *Photographic Identification of Humpback and Blue Whales off the US West Coast: Results and Updated Abundance Estimates from 2008 Field Season*. http://oai.dtic.mil/oai/oai?verb=getRecord&metadataPrefix=html&identifier=ADA539301

Galletti Vernazzani, B., Jackson, J. A., Cabrera, E., Carlson, C. A., & Brownell, R. L. (2017). Estimates of abundance and trend of Chilean blue whales off Isla de Chiloe, Chile. *PLoS ONE*, *12*(1), 1–16. https://doi.org/10.1371/journal.pone.0168646

Gendron, D., & Ugalde De La Cruz, A. (2012). A new classification method to simplify blue whale photo-identification technique. *Journal of Cetacean Research and Management*, *12*(2), 79–84.

Irvine, L. M., Winsor, M. H., Follett, T. M., Mate, B. R., & Palacios, D. M. (2020). An at-sea assessment of Argos location accuracy for three species of large whales, and the effect of deep-diving behavior on location error. *Animal Biotelemetry*, *8*(1), 20. https://doi.org/10.1186/s40317-020-00207-x

Oestreich, W. K., Abrahms, B., McKenna, M. F., Goldbogen, J. A., Crowder, L. B., & Ryan, J. P. (2022). Acoustic signature reveals blue whales tune life-history transitions to oceanographic conditions. *Functional Ecology*, *36*(4), 882–895. https://doi.org/10.1111/1365-2435.14013

Palacios, D. M., Irvine, L. M., Machette, T., Lagerquist, B. A., Falcone, E. A., Schorr, G. S., & Mate, B. R. (2025). Dispersal, revisitation, and residency of satellite-tagged blue and fin whales in the eastern North Pacific Ocean. *Animal Biotelemetry*. https://doi.org/10.1186/s40317-025-00439-9

Sears, R., Williamson, M., Wenzel, F., Bérubé, M., Gendron, D., & Jones, P. (1990). Photographic identification of the blue whale (Balaenoptera musculus) in the Gulf of St. Lawrence, Canada. *Rep IWC Spec Issue*, (12), 335–342.

Whittome, G., Calambokidis, J., Douglas, A. B., Fishbach, M., Sears, R., & Hammond, P. S. (2024). Changes in blue whale survival and abundance in the Gulf of California. *Marine Mammal Science*, *40*(4), e13132. https://doi.org/10.1111/mms.13132
